# Changes in circulating endothelial progenitor cells predict responses of multiple myeloma patients to treatment with bortezomib and dexamethasone

**DOI:** 10.1590/1414-431X20154558

**Published:** 2015-06-23

**Authors:** L. Wang, F. Du, H.M. Zhang, W.J. Zhang, H.X. Wang

**Affiliations:** 1 Department of Hematology, The Central Hospital of Wuhan, Tongji Medical College, Huazhong University of Science and Technology, Wuhan, China; 2 Department of Gastroenterology, Union Hospital, Tongji Medical College, Huazhong University of Science and Technology, Wuhan, China

**Keywords:** Chemotherapy, Circulating endothelial progenitor cells, Multiple myeloma

## Abstract

Four cycles of chemotherapy are required to assess responses of multiple myeloma (MM) patients. We investigated whether circulating endothelial progenitor cells (cEPCs) could be a biomarker for predicting patient response in the first cycle of chemotherapy with bortezomib and dexamethasone, so patients might avoid ineffective and costly treatments and reduce exposure to unwanted side effects. We measured cEPCs and stromal cell-derived factor-1α (SDF-1α) in 46 MM patients in the first cycle of treatment with bortezomib and dexamethasone, and investigated clinical relevance based on patient response after four 21-day cycles. The mononuclear cell fraction was analyzed for cEPC by FACS analysis, and SDF-1α was analyzed by ELISA. The study population was divided into 3 groups according to the response to chemotherapy: good responders (n=16), common responders (n=12), and non-responders (n=18). There were no significant differences among these groups at baseline day 1 (P>0.05). cEPC levels decreased slightly at day 21 (8.2±3.3 cEPCs/μL) *vs* day 1 (8.4±2.9 cEPCs/μL) in good responders (P>0.05). In contrast, cEPC levels increased significantly in the other two groups (P<0.05). SDF-1α changes were closely related to changes in cEPCs. These findings indicate that change in cEPCs at day 21 in the first cycle might be considered a noninvasive biomarker for predicting a later response, and extent of change could help decide whether to continue this costly chemotherapy. cEPCs and the SDF-1α/CXCR4 axis are potential therapeutic targets for improved response and outcomes in MM patients.

## Introduction

Bortezomib plus dexamethasone has been recommended as a primary therapy for multiple myeloma (MM) stem cell transplant and non-transplant candidates. After four 3-week cycles of the regimen, the rate of complete response (CR)/near CR was 14.8%, the rate of achieving at least a very good partial remission (VGPR) was 37.7%, and the overall response rate was 78.5% (1,2). However, four cycles are needed to assess a response to this valuable but costly regimen. Therefore, it would be particularly valuable in developing countries to identify a biomarker that could predict the possibility of a response in the later phase of treatment. In recent years, circulating endothelial progenitor cells (cEPCs) have been documented to change during anti-angiogenic therapy, and cEPC levels were thought to be useful as a predictive marker for monitoring chemotherapy, particularly with anti-angiogenic therapies for some cancers, such as breast cancer, non-small-cell lung cancer, prostate cancer, and MM (3 4 5 6). cEPC numbers were significantly increased in MM patients and correlated with serum β_2_-microglobulin levels (7). After a median follow-up of 4 months, the responders demonstrated a significant decrease in cEPC numbers compared to their pretreatment values (5). In addition to cEPCs, angiopoietins and a novel panel of proteins were also considered as potential predictive biomarkers for treating MM (8,9). However, these biomarkers were not studied in treatments with bortezomib. In particular, some thalidomide non-responders successfully achieved CR/VGPR using bortezomib; thus, it is debatable whether these potential biomarkers are still efficacious. The changes in cEPC levels after 7 days of chemotherapy correlated with the tumor volumes after three cycles of chemotherapy and predicted progression-free survival (PFS) or overall survival (OS), regardless of the chemotherapy regimens used with some tumors. Until now, MM biomarkers have been measured prior to therapy or after four cycles of chemotherapy. However, the values in the early phase of the first cycle of chemotherapy may be more predictive.

In this study, we specifically focused on whether changes in cEPC levels in the early phase of the first cycle of chemotherapy could predict the response of newly diagnosed MM patients to a regimen of bortezomib and dexamethasone. Because stromal cell-derived factor-1α (SDF-1α) plays a key role in both the release and homing processes of cEPCs, we attempted to determine whether it also correlated with the cEPC changes observed during chemotherapy. This study could potentially assist physicians in choosing a bortezomib-based treatment, thereby avoiding other ineffective and costly treatments; it would be particularly valuable for patients in developing countries.

## Material and Methods

### Study population

A total of 50 newly diagnosed MM patients were registered at our hospital between May 2009 and November 2014. In the end, 46 patients were available after a 4-month period to assess the response. They underwent four 21-day cycles of bortezomib (Velcade, Millennium Pharmaceuticals, USA) plus dexamethasone (n=46): bortezomib (1.3 mg/m^2^) intravenous bolus on days 1, 4, 8, and 11, and dexamethasone (20 mg) on days 1, 2, 4, 5, 8, 9, 11, and 12. For each patient, a baseline peripheral blood (PB) sample was drawn prior to therapy, and then PB samples were collected on days 7, 14, and 21. Routine laboratory data including serum concentrations of β_2_-microglobulin, M-protein, albumin, and International Staging System (ISS) stages were obtained from medical records. Thirty-five patients had adverse events such as constipation, peripheral neuropathy, thrombocytopenia, leucopenia, and secondary high glucose levels. The study was approved by the Ethics Committee of the Central Hospital of Wuhan. All patients provided signed informed consent prior to the study.

### Inclusion and exclusion criteria

The inclusion criteria included age (18-65 years) and a diagnosis of MM with the International Myeloma Working Group diagnostic criteria. The exclusion criteria were a history of inflammatory or infectious cardiovascular or autoimmune disease, use of steroids prior to the start of therapy, use of erythropoiesis-stimulating agents or granulocyte-colony stimulating factor (G-CSF), and infectious diseases such as herpes zoster. Among the 50 patients who participated in the study, 2 patients did not complete four cycles of chemotherapy and 2 patients who suffered from herpes zoster were excluded.

### Enumeration of cEPCs by flow cytometry

Using EDTA as an anticoagulant, peripheral venous blood was collected from the MM patients at four time points during the first cycle of chemotherapy. The first day of the first cycle of chemotherapy was considered to be day 1. Samples were collected in the mornings on days 1, 7, 14, and 21 (the day prior to the start of the second cycle of chemotherapy). cEPCs were measured twice at each time point, and the average was used for evaluation. PB mononuclear cells from anticoagulated blood were separated using Ficoll (BD Biosciences, USA). The tubes were centrifuged at room temperature for 20 min at 1800 *g* and then washed twice with PBS. Mononuclear cells (5×10^6^) were labeled with preconjugated mouse anti-human monoclonal antibodies: CD34 conjugated to allophycocyanin, CD309 (VEGF receptor-2 [VEGFR2]/kinase insert domain-containing receptor [KDR]) to fluorescein isothiocyanate (BD Biosciences, USA), and CD45 to peridinin chlorophyll protein-cyanine for 20 min at room temperature. The mixture was then incubated at 37°C for 30 min in the dark, washed with PBS, and then suspended in 1% paraformaldehyde and maintained at 4°C prior to analysis. cEPCs were evaluated by four-color flow cytometry (BD FACSAria II) and were identified as CD45-/dim cells with coexpression of CD34 and CD309. Although there are many methods (using different antibodies) for identifying cEPCs, CD34 and CD309 double-positive cells were considered to be cEPCs, an approach that is supported by many biological and methodological studies (10 11 12). The number of cells per milliliter of blood was calculated and compared to the mononuclear cell count of the original sample.

### Quantification of serum levels of SDF-1α by ELISA

All PB samples were collected in potassium-EDTA or serum tubes. SDF-1α levels were determined in PB samples using a commercially available SDF-1α ELISA kit (BD Biosciences) according to the manufacturer’s instructions.

### Assessment of response

Treatment response was assessed according to the international uniform response criteria for multiple myeloma (13). Briefly, complete response (CR) was defined as an undetectable level of serum M-protein and ≤5% bone marrow plasma cells (BMPCs), VGPR was defined as a reduction in serum M-protein by at least 90% of the initial value, and partial response (PR) was defined as a reduction in M-protein by approximately 50-90%. Patients with a reduction of ≤50% M-protein were defined as having stable disease (SD), and those with either an increase in M-protein of ≥25% from baseline or an increase in BMPCs of ≥10% were defined as having progressive disease (PD). Patients who achieved PR or better response were considered responders. Patients who achieved SD, PD, or a worse effect were considered non-responders.

### Statistical analysis

Continuous variables are reported as means±SD. Categorical variables are reported as number or percentage. Statistical comparisons were performed using the independent-samples and paired-samples *t*-test. Paired-samples *t*-test was used to estimate statistically significant differences in cEPC numbers between patients in different groups. The correlation between cEPC numbers and SDF-1α was evaluated using a Pearson correlation test when the data were normally distributed. All results were analyzed using SPSS 15.0 (SPSS, Inc., USA), and P<0.05 (two-sided) was considered to be statistically significant.

## Results

### Patient characteristics

Fifty MM patients were enrolled in the study; however, 4 patients did not complete the four chemotherapy cycles and, therefore, 46 patients were available for the analysis of changes in cEPCs and SDF-1α after chemotherapy. Patients were assigned to one of two groups, according to their responses. Eighteen patients fulfilled the criteria of PR or better responses and were considered to be the responders, while the remaining 28 patients who achieved SD, PD, or more serious effects were considered to be the non-responders. There were no significant differences between the two groups in terms of factors such as age, gender, M-protein, β_2_-microglobulin, serum albumin, and ISS stage. The patient demographics and clinical staging are shown in Table 1.

**Table:**
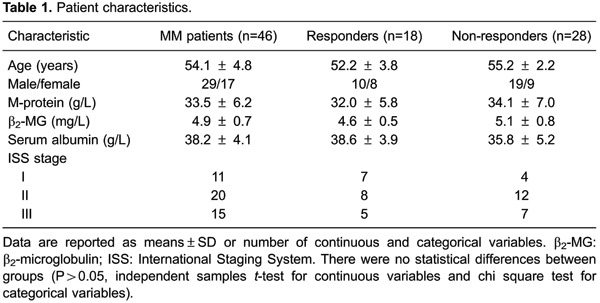

### cEPC changes during the first cycle of chemotherapy

**Figure 1:**
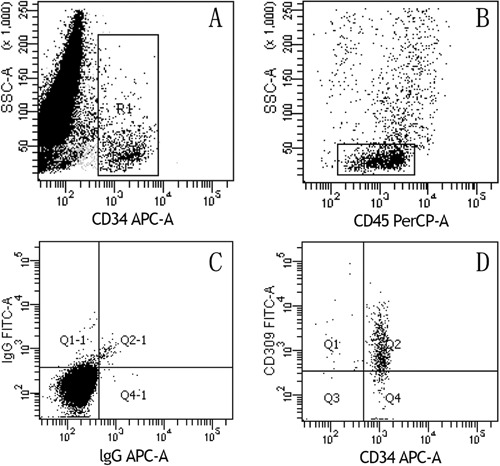
Representative flow cytometric analysis plots showing sequential gating strategy used to enumerate circulating endothelial progenitor cells (cEPCs): CD34+ and CD45-/dim cells were gated to exclude hematopoietic cells expressing CD45 antigen (*A*,*B*). *C*, Negative control, and *D*, cells co-expressing CD34 and CD309 were designated as cEPCs.

**Figure 2:**
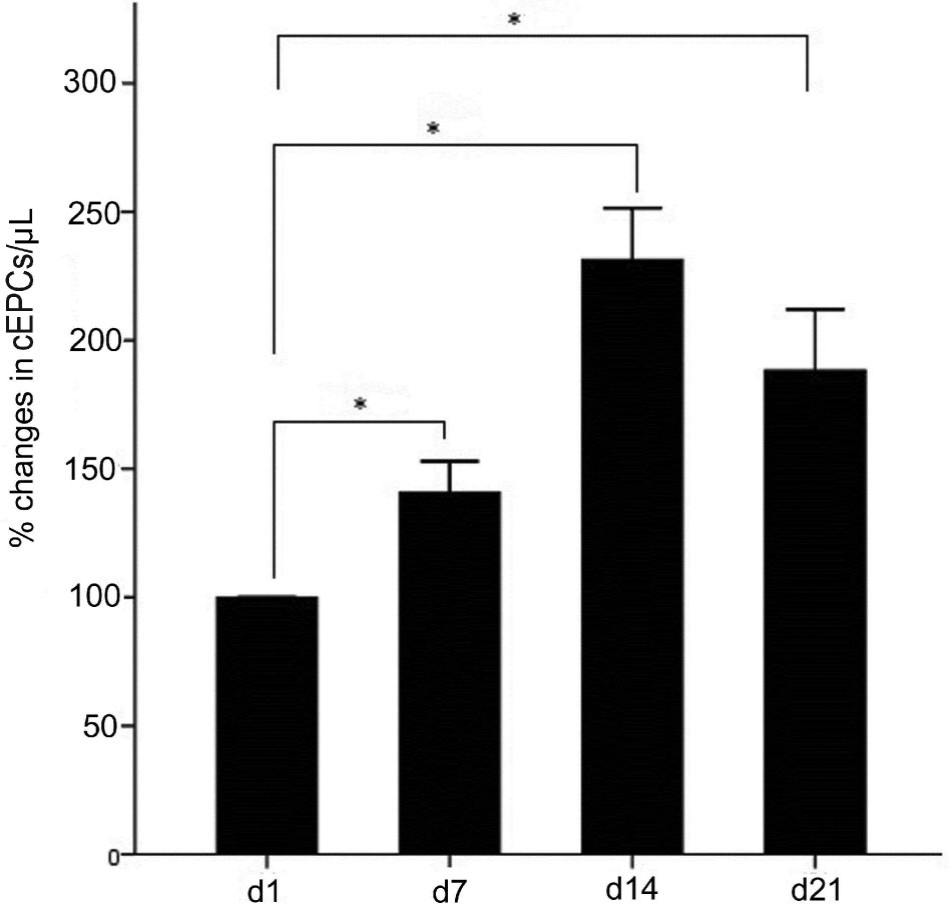
Kinetics of circulating endothelial progenitor cells (cEPCs) in the first cycle of chemotherapy. d: day. *P<0.01 (paired-samples *t*-test).

Overall, an increase was observed in cEPCs after the first cycle of chemotherapy. After 7 and 21 days, the increase in cEPCs was substantially greater than that at the beginning, and they were consistently present. At day 7, cEPC levels increased to 141% (95%CI=128-154%, P<0.01). At day 14, cEPC levels further increased to 231% (95%CI=210-253%, P<0.01). Then, at day 21, cEPC levels decreased to 188% (95%CI=163-213%, P<0.01), as shown in Figures 1 and 2.

### SDF-1α changes in the first cycle of chemotherapy

**Figure 3:**
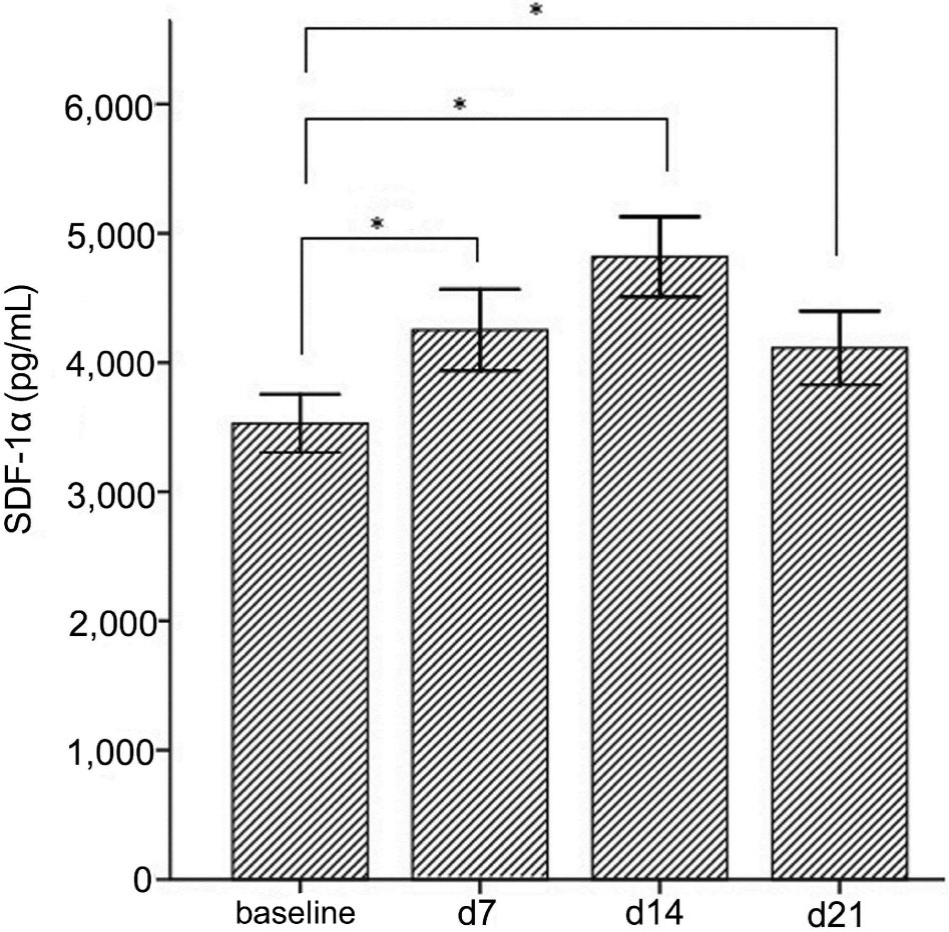
Kinetics of stromal cell-derived factor (SDF)-1α changes in the first cycle of chemotherapy. d: day. *P<0.01 (paired-samples *t*-test).

As shown in Figure 3, at baseline, SDF-1α was 3530 pg/mL (95%CI=3292-3768 pg/mL). After 7 and 14 days, SDF-1α levels steadily increased to 4253 pg/mL (95%CI=3920-4586 pg/mL, P<0.01) and 4819 pg/mL (95%CI=4491-5148 pg/mL, P<0.01). At day 21, SDF-1α levels decreased to 4112 pg/mL (95%CI=3811-4414 pg/mL, P<0.01) but remained higher than the baseline level. There was a significant positive correlation between cEPC and SDF-1α at 7 days (Pearson r=0.48, P=0.02), 14 days (Pearson r=0.53, P=0.01), and 21 days (Pearson r=0.482, P=0.02) after chemotherapy.

### Changes in cEPC levels and response

**Figure 4:**
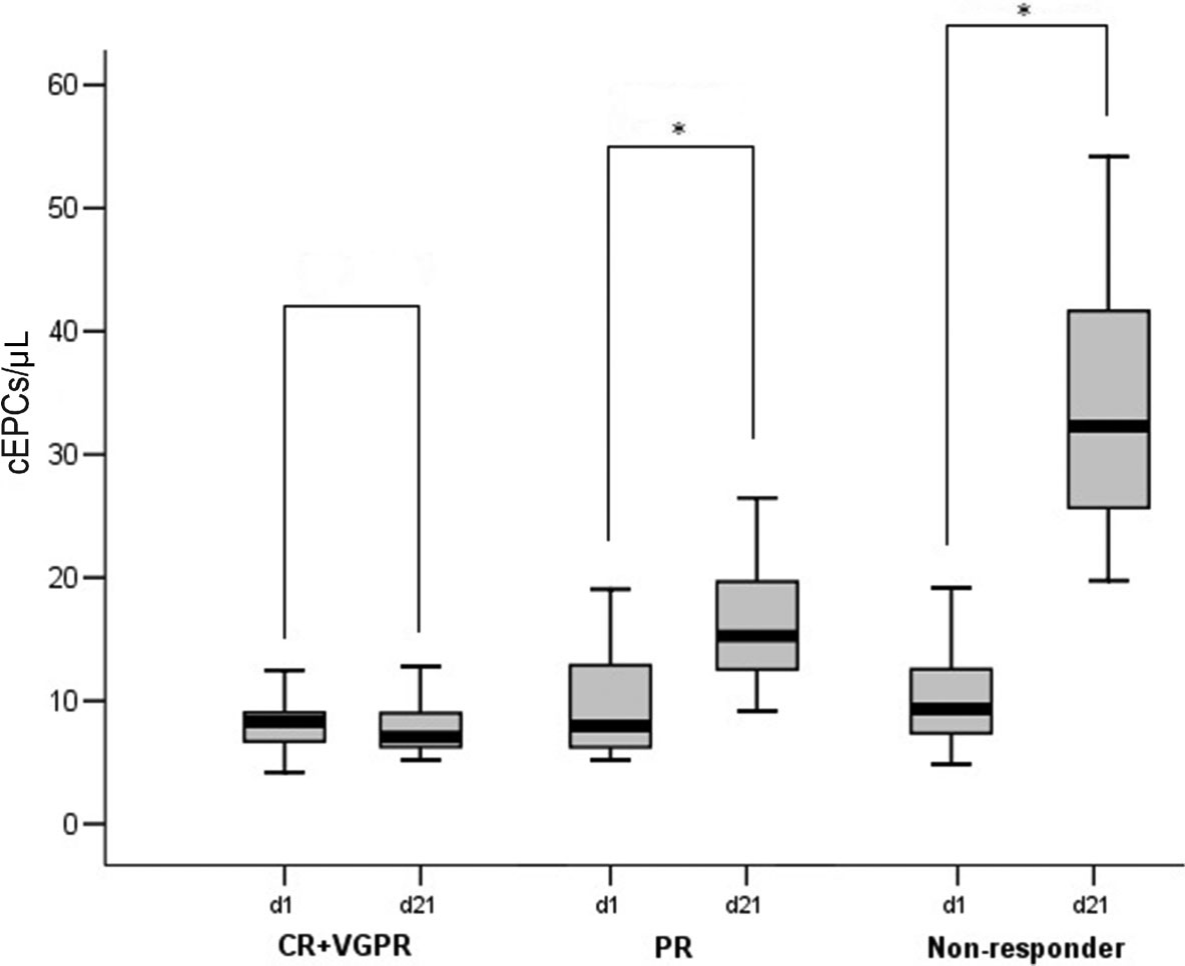
Box-plot representation of the circulating endothelial progenitor cells (cEPCs) levels at days 1 and 21. Box-plots show median (middle line), interquartile range (box), 25-75th percentile (whiskers). CR: complete response; VGPR: very good partial remission; PR: partial response; d: day. The paired-samples *t*-test was used for statistical analyses.

After four cycles of chemotherapy, CR was achieved in 5/46 patients (10.9%), VGPR in 11/46 patients (23.9%), and PR in 12/46 patients (26.1%); and 18/46 patients (34.8%) had SD or PD. The study population was divided into 3 groups according to the response to chemotherapy: good responders (CR+VGPR, n=16), common responders (PR, n=12), and non-responders (SD+PD, n=18). There were no significant differences among these 3 groups at the baseline day 1 (chi-square=2.169, P=0.338), and cEPC numbers in the 3 groups were (means±SE) 8.4±2.9, 9.8±4.5, and 10.3±4.0 cEPCs/μL, respectively, as shown in Figure 4.

cEPC levels decreased minimally at day 21 (8.2±3.3) compared with day 1 (8.4±2.9) in the subgroup of good responders, but this was not statistically significant (P=0.775). In contrast, cEPC levels in the other two groups increased significantly. Within the PR group, cEPC levels increased to 16.2±5.1 from 9.8±4.5 (P=0.002), while cEPC levels increased progressively to 33.4±9.8 from 10.3±4.0 (P<0.001) within the non-responders group.

## Discussion

Bone marrow angiogenesis played an important role in the pathogenesis and progression of MM (14). It is well documented that the anti-myeloma effect of bortezomib partly occurs through a reduction of angiogenesis. Patients who responded well were most likely to have a significant decrease in microvessel density, whereas patients with stable or increased microvessel density experienced a relapse (15). The fact that angiogenin levels decreased following bortezomib treatment also suggested an anti-angiogenic mechanism. *In vitro*, bortezomib also produced an anti-angiogenic effect in MM patient-derived endothelial cells. Dexamethasone could functionally attenuate endothelial responses to VEGF, which plays an important role in the angiogenesis of MM (16). cEPCs are increased in MM patients and correlated with angiogenesis (7). In this study, we confirmed that the extent of change for cEPC numbers from day 1 to day 21 during the first cycle of treatment could predict the efficacy after four cycles. The smaller the change the more likely it is to achieve a better clinical effect. These results suggest that if an MM patient’s angiogenesis was not inhibited effectively in the first cycle of chemotherapy with this regimen, then the patient might have a poor response, even after four cycles.

In addition to targeting myeloma plasma cells, anti-myeloma drugs, such as thalidomide, lenalidomide, bortezomib, and dexamethasone, also exert direct or indirect anti-angiogenic effects. Many angiopoietins, such as basic fibroblast growth factor (bFGF) and angiopoietin-2 (Ang-2), were found to correlate with VEGF and cEPCs in MM patients; therefore, the addition of an anti-angiopoietin agent may improve the efficacy of anti-myeloma therapy (8). SDF-1α is known for its key role in both the release and homing of cEPCs (17). It has been reported that SDF-1α is increased in MM and correlated with the load and angiogenesis of myeloma plasma cells (18,19). The SDF-1α/CXCR4 axis has been shown to play an important role in neovascularization, metastasis, and chemotherapy resistance in MM. Here, we found SDF-1α was also correlated with levels of cEPCs in MM during the first cycle of chemotherapy. Conceptually, these findings point to an array of new therapeutic strategies by combining chemotherapy with agents capable of inhibiting the release of progenitor cells, such as SDF-1α/CXCR4 antagonists, which may enhance the therapeutic potential of conventional chemotherapy (20 21 22 23). Both dexamethasone and thalidomide played an indirect role in targeting the SDF-1α/CXCR4 axis (24,25). AMD3100, a small-molecule CXCR4 antagonist, has been used to collect hematopoietic stem cells for transplantation in MM patients (26). In addition, it could enhance the tumor reduction induced by bortezomib (27).

This study has several limitations. First, we lacked a long-term follow-up; therefore, we did not have the patients’ PFS and OS data for further in-depth analysis. Second, because of the small sample size, we could not provide a definite threshold for the cEPC changes necessary to predict the response.

In conclusion, this study has shown that chemotherapy evokes a host response that is composed of the release of cEPCs and SDF-1α. The extent of this release during the first cycle of chemotherapy correlates with the response four cycles later. cEPCs might be an early predictor of therapy response. Furthermore, SDF-1α might be the key reason for cEPC changes, and targeting SDF-1α/CXCR4 axis therapy or combining it with conventional chemotherapy could improve the responses and outcomes of MM patients.
